# Emerging Roles of Cytoneme-Mediated Signaling in Cancer

**DOI:** 10.3390/ijms27177600

**Published:** 2026-08-25

**Authors:** Sheikh Faisal Asadullah Mahdi, Eric T. Hall

**Affiliations:** Department of Human Anatomy and Cell Science, University of Manitoba, Winnipeg, MB R3E 0J9, Canada

**Keywords:** cytoneme, cancer signaling, myosin 10, tumor microenvironment, cell–cell signaling, morphogens, cytoskeleton

## Abstract

Intercellular communication across cancer cells and the tumor microenvironment (TME) is essential for tumor growth, invasion, metastasis, and therapeutic resistance. Traditionally, these interactions have been viewed through the lens of diffusible signaling molecules and extracellular vesicles. However, growing evidence supports an additional paradigm in which specialized cytoskeleton-based membrane extensions, like tunneling nanotubes (TNTs), tumor microtubes (TMs), and cytonemes, mediate direct, contact-dependent communication between cells. This review examines the emerging roles of these cellular extensions in cancer biology, with a particular emphasis on cytonemes, long specialized signaling filopodia that facilitate transport and reception of signaling ligands and receptors. Cytonemes are interwoven with developmental signaling pathways, which are frequently reactivated in cancer, promoting tumor progression. We discuss cytoneme pathology in cancer, with specific examples in growth, stemness, invasion, and microenvironmental remodeling. These extensions represent an unexplored facet of tumor biology and a promising avenue for therapeutic intervention.

## 1. Introduction

### Intercellular Cancer Signaling Beyond Diffusion

Although most cancers arise from intrinsic genetic and epigenetic alterations within tumor cells, interactions between malignant cells and the surrounding tumor microenvironment (TME) are equally important determinants of disease progression. Tumor growth, invasion, metastasis, and therapeutic resistance arise not only from cell-autonomous changes, but also from dynamic reciprocal signaling between cancer cells and surrounding stromal, vascular, and immune populations. This continuous evolution between cancer cells and the surrounding TME involves extensive remodeling of the extracellular matrix reorganization, angiogenesis, recruitment of cancer-associated fibroblasts (CAFs), immune-cell infiltration, and metabolic adaptation (reviewed in [1,2,3]). These processes are coordinated through complex networks of intercellular communication governed by a myriad of different signaling molecules being exchanged between the tumor and stroma. These signaling molecules include growth factors, cytokines, chemokines, and morphogens. Historically, these molecules have been thought to be sent through the extracellular space by mechanisms like diffusion and transcytosis, either freely or through extracellular vesicles and exosomes, eventually binding and activating receptors and responses on nearby cells to mediate tumor–stroma communication [3,4].

A continually growing body of evidence is highlighting an emerging paradigm with cells establishing direct communication networks through specialized cytoskeleton-derived membrane extensions. These structures include tunneling nanotubes (TNTs), tumor microtubes (TMs), and cytonemes to create direct contact points between non-adjacent cells, allowing the transfer of signaling molecules, receptors, organelles, nucleic acids, and other cellular cargos [5,6,7]. Although these structures share the ability to connect distant cells, they differ in both composition and function. TNTs and TMs are best known for facilitating the intercellular exchange of diverse cytoplasmic cargo and electrical signals, while cytonemes are distinct in that they primarily mediate targeted signaling through the transport and presentation of morphogens and their receptors. The full extent of these differences is discussed below. Together, these extensions have now been shown to play a critical role in tumor–stroma signaling, contributing to tumor growth, therapeutic resistance, immune modulation, and microenvironmental remodeling across diverse cancer types.

Among these structures, cytonemes are of particular interest due to their well-established role in facilitating signaling for many developmental pathways, including Hedgehog (HH), WNT, Bone Morphogenetic Protein (BMP), Fibroblast Growth Factors (FGFs), and Notch [5,7,8,9]. These same pathways are frequently reactivated during tumorigenesis, yet cytonemes’ role in cancer signaling has only recently begun to be investigated. In this review, we examine the expanding roles of cytoskeletal communication networks in cancer, with a focus on cytonemes. We explore the emerging evidence of cytoneme-mediated signaling in cancers and discuss how these structures may play a previously hidden role in establishing highly localized, directional signaling, guiding tumor–stroma interaction, and dictating tumor/TME progression, as well as offer a potential highly effective therapeutic target.

## 2. Cytoskeletal Extension in Cancer and Signaling

Specialized cytoskeletal-derived membrane extensions have been widely documented in interactions between tumor cells and with the surrounding stroma [10,11,12,13,14,15]. These extensions can be classified into several categories, including lamellipodia, filopodia, invadopodia, microtentacles, cytonemes, tunneling nanotubes (TNTs), and tumor microtubes (TMs) (Table 1). Although these various actin- and/or microtubule-based extensions have defining characteristics and functions, they also share multiple conserved components. As a result, different research groups’ publications may use different terminology to describe similar structures. In general, lamellipodia, filopodia, invadopodium, and microtentacles are required for migration, environmental sensing for guidance, adhesion, and invasion [10,12,13], whereas cytonemes, TNTs, and TMs regulate signaling and communication in cancers [14,15].

## 3. Tunneling Nanotubes and Tumor Microtubes

Focusing on signaling and communication extensions, TNTs are actin-based cellular extensions that may or may not also contain microtubules [35,46]. Since they were first identified in rat adrenal medulla cells (PC12) by Rustom et al. in 2004 [38], the role of TNTs in cancer biology and signaling has grown extensively. The defining characteristic of TNTs is that they are generally an open-ended connection enabling the bidirectional exchange of intracellular components, including soluble proteins; nucleic acids; vesicles; organelles, viruses, and other cytoplasmic components between cells [15,37]. TNTs can connect cells that are hundreds of microns apart, and they typically range between 150 and 1000 nm in diameter [47]. The lifespan of these intercellular connections varies depending on cell type, ranging from minutes (~30 min) [48] to over 24 h in more stable microtubule-containing TNTs [49]. The exchange of cytoplasmic components through TNTs supports intercellular stress mitigation and cell survival, with mitochondrial transfer demonstrated to be a key component of this [50,51,52]. As cellular stress adaptation and response are central drivers of tumor progression [53], the roles and contributions of TNTs in cancer-cell survival and the TME have come to the forefront of research in recent years. Importantly, TNTs not only establish communication networks between tumor cells; they also contribute to the development of a tumor-supportive microenvironment by connecting malignant cells with surrounding non-malignant stromal and immune cells. Through these heterotypic interactions, TNTs have been shown to facilitate the resistance of tumor cells against treatments, including surgery, radiotherapy, and chemotherapy [40,42,54], as well as facilitate immune-evasion [51]. These findings have been extensively discussed and highlighted in other recent reviews [6,15,41,55].

TMs play a similar role to that of TNTs by facilitating tumor-cell survival and resistance to therapies, by transporting similar cellular components [44]. Unlike the predominantly actin-based TNTs, which persist for minutes to possibly days [48,49], the generally thicker actin- and tubulin-stabilized TMs are considered more robust and long-lived, lasting from days to weeks [42,43]. TMs are frequently identified in gliomas, where they serve as key mediators of calcium and small-molecule exchange between glioma cells and surrounding non-malignant cells of the TME through Connexin 43 positive gap junctions [42]. Tumor-cell TMs and TNTs’ connections also facilitate the building of synaptic-like connections with neurons and glia, enabling exchange of neurotransmitters and promoting neuronal mimicry, not only in primary brain cancers but also in metastatic lesion that have infiltrated the brain [56,57]. With advances in microscopy, both TMs and TNTs are now being identified in a diverse range of cancers and contexts, highlighting their importance in intercellular communication, signaling, and survival [6,15,41,55]. However, due to the diversity of contexts in which these extensions are now being found, there is some ambiguity in terms of defining the essential cytoskeletal components, key molecular features, and morphology of these cellular extensions [6,58,59].

## 4. Cytonemes: Specialized Signaling Filopodia

Cytonemes are specialized, highly dynamic, actin-based signaling filopodia dedicated to contact-based communication between distant cells through the transport and reception of signaling ligands and receptors [60,61]. They were first observed in mesenchyme of sea urchin blastula in 1961 [62] and then later fully characterized in the *Drosophila* wing imaginal disc by Ramírez-Weber and Kornberg in 1999 [63]. Since then, cytonemes have become closely linked to many developmental signaling pathways and processes of embryonic development (reviewed in [5,6,7]). An initial defining characteristic distinguishing cytonemes from other filopodia was their directional orientation. Cytonemes with morphogen receptors extended primarily toward morphogen-producing cells, while cytonemes from morphogen/ligand-producing cells are directed toward receptor-expressing cells in the *Drosophila* wing imaginal disc [63]. Since this initial discovery of cytonemes’ role in Decapentaplegic (Dpp) signaling (the ortholog of mammalian Bone Morphogenetic Protein (BMP) 2 and 4) in *Drosophila* [63], cytonemes have been identified as contributors to developmental and differentiation processes, adult stem-cell niche maintenance, and repair processes of many other model organisms, including xenopus, zebrafish, chick, axolotl, and mice. Cytonemes do this by facilitating the signaling of many different core developmental pathways, including Epidermal Growth Factor (EGF) [60], Fibroblast Growth Factor (FGF) [31,60,63,64,65,66]), WNT [29,33,67,68,69,70,71,72], Notch [67,71,73,74], Hedgehog (HH) [30,71,75,76,77,78,79], and BMP signaling [63,80]. Notably, these same signaling pathways are reactivated and exploited in diverse cancers to drive proliferation, stemness, invasion, and metastasis, while also being frequently targeted therapeutically (reviewed in [81,82,83,84]). The close relationship between cytoneme and these developmental/cancer-associated signaling pathways makes cytonemes an exciting potential candidate for novel therapies in cancer treatment. However, research into this area is in its infancy, with direct evidence of cytoneme-mediated signaling in cancer currently limited to a few recent studies [14,32,33,34].

The lack of direct evidence of cytonemes in intercellular cancer signaling likely stems from two factors to date. First, this may be in part due to the misidentification of some cytonemes as TNTs, due to the overlapping and heterogeneous nature of TNTs outlined in the current literature [58]. Second, and likely the major contributor, cytonemes are exceptionally thin and fragile structures, making preservation and detection of cytonemes difficult outside of live-imaging conditions. Cytonemes are ~70–200 nm in diameter, with closed-ended actin-based extension, and capable of growth up to several hundred microns in length [5]. Additionally, cytonemes are more dynamic and transient than TNTs: they extend and contact other cell bodies or cytonemes on a scale of seconds to minutes before releasing contact [9]. During this short time, they form synapse-like connections, allowing signal delivery and reception [5,85]. As such, cytonemes’ biology has predominantly been carried out in developing embryos, where these structures can be detected more easily under live-imaging conditions. Furthermore, standard fixation approaches fail to effectively preserve cytonemes, limiting their detection in postnatal tissues, and tumors. More recently, specialized preservation approaches, like Modified Electron Microscopy Fixation (MEM-fix), have greatly enhanced the capacity to preserve and study cytonemes in fixed cells and embryos [86,87,88,89]. However, effective preservation and detection in postnatal tissue and tumor specimens remain a major technical hurdle, limiting our understanding of their prevalence and function in cancer.

## 5. Cytoneme Initiation and Regulation

As specialized filopodia, cytonemes are regulated by many of the conserved actin-cytoskeletal components found in filopodia formation and maintenance. Initiation of cytoneme outgrowth occurs predominantly through activation of the Rho GTPase family member Cdc42 [29,74]. Once activated, Cdc42 interacts with downstream effectors, commencing a cascade of actin nucleation and polymerization through proteins like Wiskott–Aldrich syndrome protein (N-WASP) and membrane remodeling proteins like IRSp53 [29]. Fascin-bundled actin-filament cores require diaphanous-related formins for actin polymerization to drive elongation and outgrowth [5,80,90].

The mechanisms of initiation, and likely the corresponding prolonged growth cues, also distinguish cytonemes from other actin-based extensions. Notably, cytoneme formation has been extensively linked to the same morphogens, growth factors, and receptors that they transport. In Hedgehog (HH) signaling, pathway ligands, release proteins, and co-receptors have all been identified to promote cytoneme formation, thereby reinforcing and increasing signal transduction across tissues [30,71,77,89,91]. Similar relationships have been shown with components of the WNT [29,68,69,70], Notch [30,67,74], and FGF signaling pathways [30,64,65]. Thus, many fundamental developmental signaling pathways that are frequently reactivated during tumorigenesis are also fundamentally coupled to enhancing cytoskeleton regulation and cytoneme formation and function, potentially enhancing the delivery of signaling factors that contribute to tumor–stroma signaling.

This mechanism of signal transport varies among pathways and can even differ depending on different ligands for the same pathway. In WNT signaling, WNT5a/b and WNT8a ligands are thought to accumulate on the outer plasma membrane surface with receptor ROR2 to drive cytoneme outgrowth and transport to receiving cells [68,69,70]. However, WNT8a has also recently been described to be transported within cytonemes in vesicles [92], suggesting possible cell-specific or context-dependent mechanisms of initiation versus continued delivery. A similar outer membrane mode of transport has been reported for the FGF homolog, Branchless (Bnl), in *Drosophila* [64]. In contrast, HH ligands are internalized, along with the HH release protein DISP and co-receptors BOC/CDON as a complex, and transported internally within cytonemes in small exovesicles to be released at cytonemes’ tips [30,71,76]. This coupling of intercellular signaling pathways and cytoskeletal regulation highlights cytonemes as an excellent potential therapeutic target. Therapeutically disrupting cytoneme formation or function can simultaneously impair multiple oncogenic signaling pathways and effectively suppress several cancer-promoting mechanisms concurrently.

## 6. Myosin 10 at the Nexus of TNTs, Cytonemes, and Cancer Progression

Although mechanisms of cytoneme initiation and entry are partially pathway-dependent for different signaling ligands and receptors, vertebrate cells appear to use similar mechanisms of protein transport to reach the cytonemes’ tip. The unconventional actin-motor protein Myosin 10 (Myo10) plays an essential role in filopodia and cytoneme maintenance and growth, as well as signal-protein transport [30,93,94,95]. Functional Myo10 proteins form as dimers consisting of three distinct regions. The head domain acts as the actin-based motor, binding to actin filaments to “walk” along them. The neck region, which consists of three IQ motifs, can bind to a calmodulin or calmodulin-like light chain, acting as a lever arm to regulate step size and motor activity [94]. The tail region consists of a single alpha helix region and a coiled-coil domain contributing to dimerization, followed by PEST, PH, MyTH4, and FERM domains [94]. The PEST domain is important for regulating protein stability [96]. The three clustered PH domains (specifically PH2) allow direct interactions with phosphatidylinositol (3,4,5)-trisphosphate (PIP3) in the plasma membrane of cells, activating Myo10 while simultaneously enriching Myo10 to filopodia and promoting both cytoneme and neuronal TNT formation [30,97,98,99]. The MyTH4 domain binds to microtubules, allowing Myo10 to act as a motorized link between the actin and tubulin cytoskeleton [100]. The combined MyTH4-FERM domain of Myo10 has been shown to bind to many cargos, such as transmembrane proteins, including integrins and various receptors [94]. Through these domains, Myo10 can interact with membrane lipids and transmembrane proteins while moving toward the tips of bundled actin filaments, recruiting proteins, and generating force for continued actin polymerization [101]. This makes Myo10 an essential protein for actin-based cytoskeleton outgrowth and signaling in cytonemes and some TNTs [30,71,102]. Myo10 has been shown to colocalize with various WNTs in cytonemes and to carry WNT8a- and SHH-containing vesicles along cytonemes for transport and subsequent release at cytoneme tips [30,34,70,92]. Loss of Myo10 decreases cytoneme formation, impairing SHH, WNT, and Notch activity in developing mice embryos [71]. Interestingly, *Drosophila* does not have an overt Myo10 homolog, suggesting that different molecular motors may facilitate this transport role, possibly through the myosin XV homolog, *Sisyphus* [103].

Myo10 has been shown to play a multifaceted role in many cancers, particularly in promoting metastasis through its role driving various actin-based extensions [95], but also controlling aspects of genomic stability [104]. Elevated Myo10 expression is seen in a wide range of cancers and frequently corresponds with poorer prognosis. This includes melanomas [105,106], breast cancer [105,107,108], cervical cancer [109], leukemias [110], lung cancers [111,112], prostate cancer [113], and glioblastomas [114]. Myo10 mRNA expression can be used as a prognostic marker for survival in patients with squamous-cell lung carcinoma [112]. Myo10 has been shown to drive melanoma development and metastasis through the formation of long cytoneme-like extensions and cell migration [106]. Many cancers use invadopodia to penetrate basal membranes for invasion and metastasis. Knocking out Myo10 limits invadopodia elongation and formation, hampering matrix degradations and invasion [115]. Additionally, knockdown of Myo10 decreases filopodium-like protrusions (FLPs) in invasive mammary carcinomas [23]. Reduced FLPs decrease metastatic colonization and cell proliferation, as FLPs stimulate FAK/ERK signaling to drive this process. In glioblastoma mouse models, Myo10 knockout decreases GBM invasion, proliferation, and integrin signaling, increasing lifespan and survival time [114]. Similar effects are seen in colorectal cancers, with Myo10 knockout inhibiting metastasis and proliferation through a RACK1/integrin/Src/FAK signaling cascade [116]. Conversely, elevated Myo10 expression levels correspond to increased invasion and metastasis in multiple breast cancer subtypes, through its role in invadopodia formation and integrin receptor transport to filopodia tips [105,107,108]. Myo10 may also play a prominent downstream role in PTEN/PI3K-driven cancer progression [117]. Tumors with PTEN mutation are linked with aberrant PIP3 generation, which can drive Myo10 activation [99,118].

Beyond Myo10’s essential role in promoting actin-based cellular extensions, it also contributes to regulating cell division and genomic stability [104]. Through tail interaction with microtubules, Myo10 regulates mitotic spindle structure and dynamics, functions, orientation, and bipolarity [119,120,121,122]. A high level of Myo10 in the nucleus can disrupt the nuclear cytoskeleton network balance, causing abnormal morphology at the perinuclear space and thereby driving genomic instability during mitosis and inflammatory response [104]. This can cause context-dependent differences, either promoting cancer progression through elevated mutation rate or inhibition of cell cycle.

With increasing evidence that cellular protrusions and cytoskeletal proteins play a central role in cancer biology, Myo10 has come to the forefront at an intersection between supporting invasion, migration, and signal transduction to drive cancer progression. Together, these functions suggest that MYO10 is an important contributor to cancer biology and a strong potential therapeutic target. However, MYO10 contributes to numerous post-developmental physiological processes beyond cancer, including filopodia formation, cell migration, axon guidance, wound healing, and mitotic spindle regulation [94,95,123]. As a result, systemic suppression of MYO10 activity could potentially impair normal tissue homeostasis and regenerative responses, particularly impacting tissues where developmental signaling pathways remain active in adults. This would affect stem-cell niches with long-term inhibition that is likely to influence proliferative tissues with high rates of cell turnover.

In contrast to these potential limitations, germline loss of Myo10 in mice still generates viable and fertile animals [124]. Additionally, Myo10 deletion enhances the DNA damage response in murine and human GBM cells, but not in normal tissues [114]. The dependence of many tumors on elevated MYO10 activity may provide a therapeutic window in which malignant cells are more sensitive to MYO10 inhibition than normal tissues. However, defining this therapeutic index and identifying potential toxicities will be essential before MYO10-targeted therapies can be translated to the clinic.

## 7. Cytoneme Based Signaling in Cancer

Although cytonemes were first characterized as developmental signaling structures, recent evidence demonstrates that tumors co-opt cytoneme-mediated signaling to coordinate communication between cancer cells and the TME [14,32,33,34]. These studies have visualized cytonemes and tested their functional importance in different cancer models, demonstrating that cytonemes are active participants in tumor growth, invasion, and niche remodeling rather than passive cellular protrusions [14,32,33,34].

**Figure 1:**
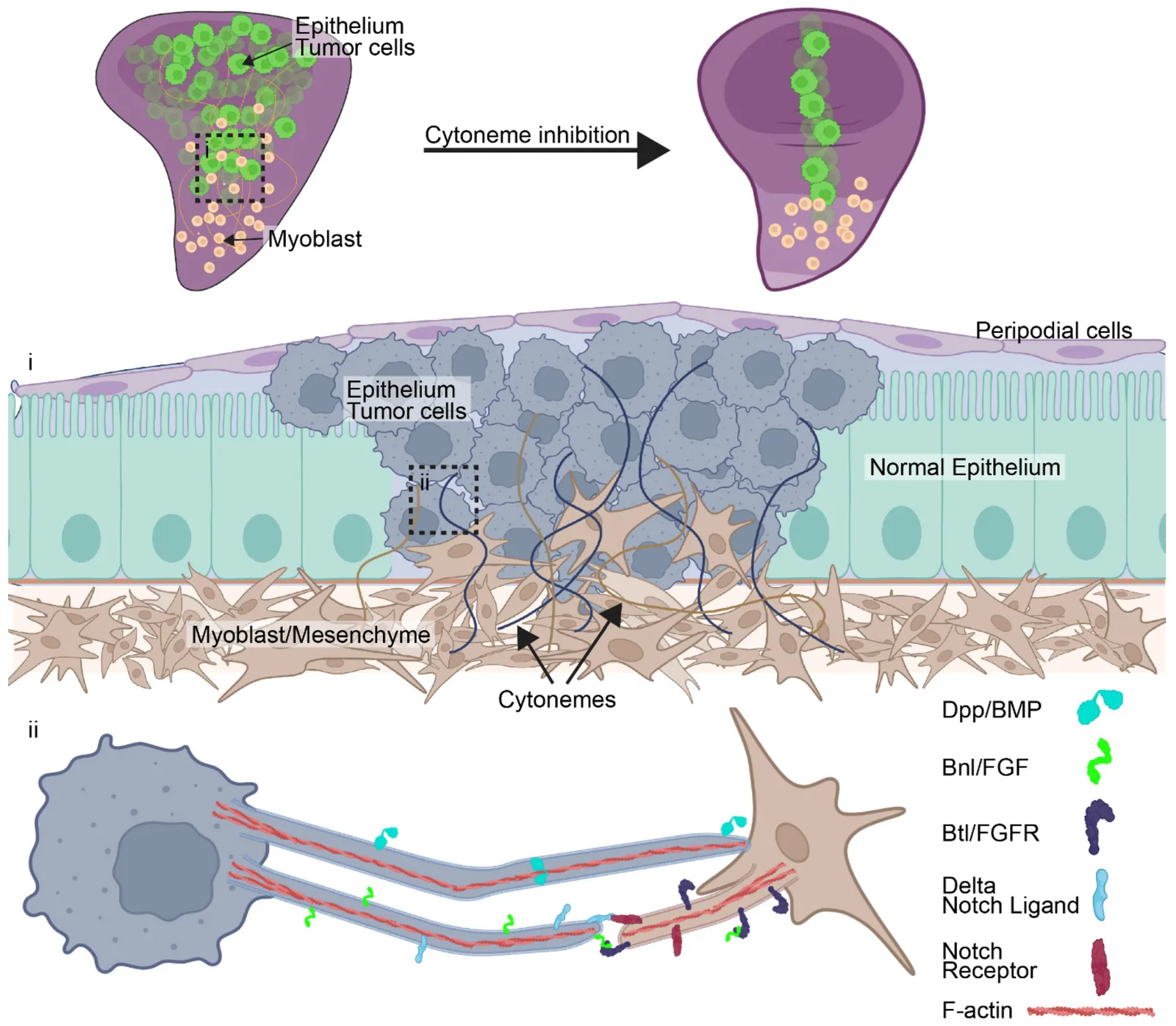
Cytoneme signaling in *Drosophila* tumor models. EGFR- and RET-driven epithelium tumor models in the *Drosophila* wing imaginal discs exhibit massive over-proliferation and invasion properties, resulting in animal death. Genetic inhibition of essential cytoneme genes in the tumor or underlying myoblasts suppresses overgrowth and lethality. (**i**) Sagittal cross-section of wing disc and with cytoneme network of interactions between tumor cells and underlying myoblast/mesenchyme and tracheal cells. (**ii**) EGFR epithelium tumor-cell cytonemes send Dpp/BMP and Bnl/FGF and Delta to underlying cells and cytonemes, activating the corresponding pathways. Activated Notch in mesenchymal cells promotes their proliferation and prevents differentiation, further supporting tumor growth. Created in BioRender. Vanan, M. I. (2026) https://BioRender.com/0izwukh.

The first example of cytoneme-mediated signaling in tumor progression came from *Drosophila* cancer models (Figure 1). Fereres et al. showed that EGFR- and RET-driven tumors depend on cytoneme-based communication between tumor cells and surrounding myoblasts and tracheal cells [14]. These cytonemes transported Dpp/BMP and FGF-pathway components to establish reciprocal signaling interactions that supported both tumor growth and remodeling of the surrounding microenvironment. Knockdown of various key cytoneme-regulating components, including Diaphanous, Neuroglian, Capricious, SCAR, and Irk2 [80,85], impaired cytoneme formation, reduced signaling activity, suppressed tumor growth, and rescued survival of tumor-bearing flies [14]. These findings provided the first direct example that cytonemes are not merely associated with tumors but are functionally required for tumorigenesis and maintenance of malignant tissues. Soon after, Boukhatmi et al. demonstrated that cytonemes act as mediators of oncogenic Notch signaling between tumors and stromal compartments in *Drosophila* [32]. Here, they identified that epithelial tumor cells extend cytonemes carrying the Notch ligand, Delta, to activate Notch signaling in adjacent mesenchymal cells (Figure 1). Activation of stromal Notch signaling promoted mesenchyme proliferation and maintenance of an undifferentiated state, generating a supportive TME that fueled tumor expansion [32]. Reducing cytoneme formation in tumor cells diminished Notch activity in the stroma and impaired tumor growth, highlighting how cytonemes facilitate bidirectional tumor–stroma signaling. This demonstrated that cytoneme-mediated signaling can allow tumors to actively reprogram neighboring cell populations to support disease progression.

**Figure 2:**
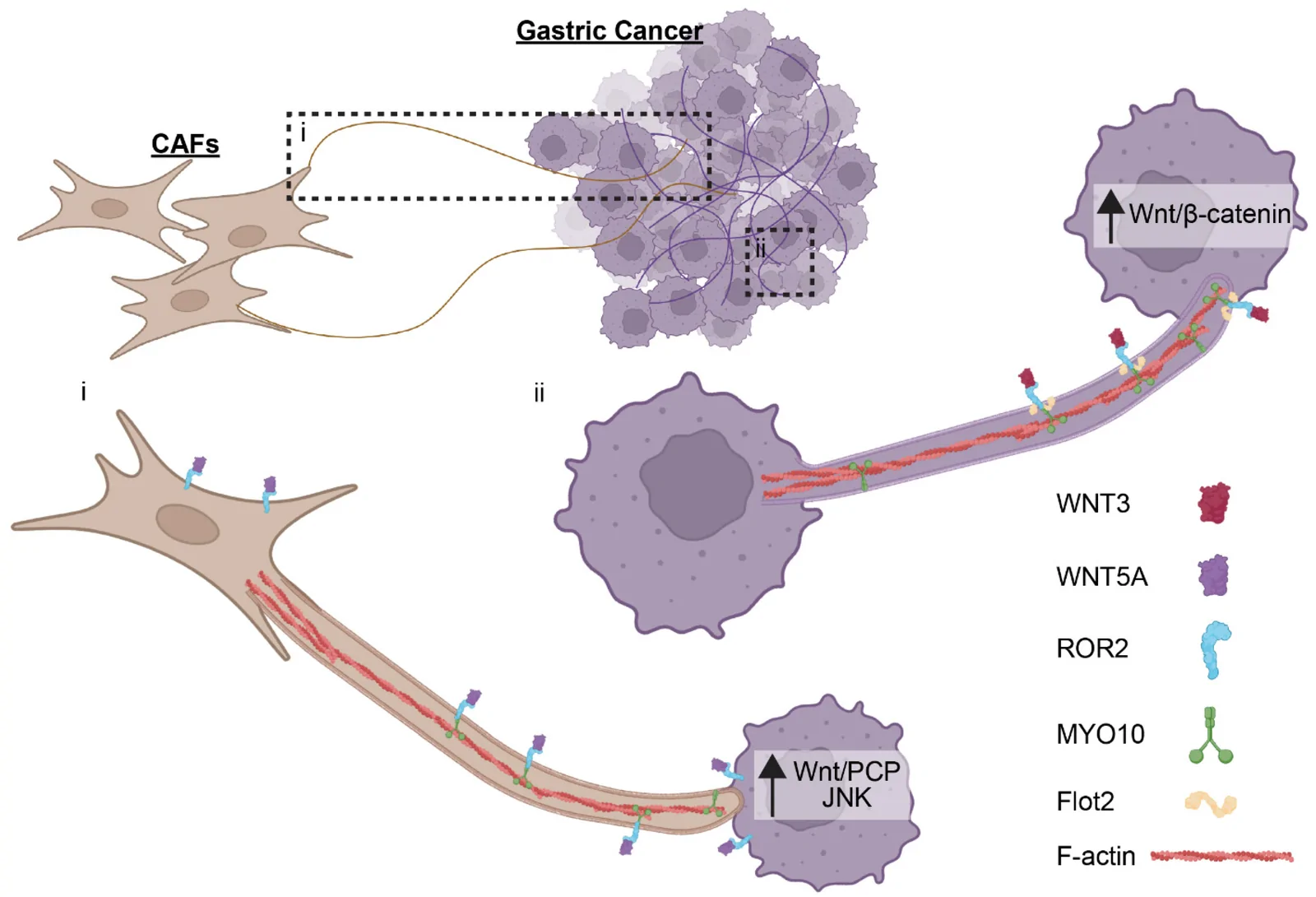
Cytonemes mediate WNT signaling to promote gastric cancer proliferation and migration. Gastric cancer cells and supporting CAFs use cytonemes to facilitate (**i**) heterotypic and (**ii**) homotypic interactions to activate different WNT pathways. (**i**) CAFs produce WNT5A and receptor ROR2. Gastric cancers cells have low levels of ROR2, and therefore, they are not responsive to WNT5A alone. CAF cytonemes convey both WNT5A and ROR2 onto gastric cancers cells, allowing activation of Wnt/PCP and downstream JNK signaling driving cell polarization and migration of cancer cells. (**ii**) Gastric cancer cells produce WNT3. Flot2 promotes cytoneme elongation and numbers in cancers cells, allowing increased WNT3/ROR2 movement to activate Wnt/β-catenin signaling on nearby cells for proliferation and cell survival. MYO10 is crucial for the movement of WNT proteins along cytonemes.

Cytoneme-mediated signaling has also been demonstrated to promote proliferation in gastric adenocarcinomas (Figure 2). Work by Routledge et al. revealed that gastric cancer cells utilize cytonemes to transport WNT3 protein between cells [34]. Disruption of cytoneme formation through inhibition of IRSp53, a Cdc42 binding protein critical for filopodial formation [125], reduced WNT/β-catenin signaling, decreased proliferation of neighboring epithelial cells, and impaired cancer stem-cell colony formation [34]. The study also identified the scaffolding protein Flotillin-2 (Flot2), which is frequently overexpressed in gastric cancer, as a key regulator of WNT3-bearing cytonemes. Flot2 promoted cytoneme numbers and elongation, enhanced WNT3 dissemination, and increased paracrine signaling activity. This demonstrated that cytonemes contribute to the dissemination of an oncogenic ligand in human cancers and support tumor-promoting WNT signaling.

Further expanding cytonemes’ role in gastric cancers, Rogers et al. demonstrated that cancer-associated fibroblasts (CAFs) signal with gastric cancer cells through cytoneme-dependent transfer of the WNT and receptor ROR2 [33]. CAFs expressed elevated levels of both WNT5A and ROR2, and they generated cytonemes that contacted neighboring tumor cells. Remarkably, ROR2-containing cytoneme tips are transferred from CAFs to cancer cells, enabling cancer cells with otherwise low receptor expression to respond to the stromal WNT5A. This ligand and receptor transfer activates WNT/planar cell polarity (PCP) signaling and enhances JNK activity, promoting migration and invasion [33]. The study demonstrated that cytoneme-mediated communication transfers not only ligands, but also signaling receptors, between cells, thereby altering the signaling competence of recipient tumor cells.

These initial studies provide strong support of cytonemes’ role in human cancers. However, current published evidence for cytonemes in human-patient samples is lacking, with examples limited to gastric cancer cells, HEK293T, induced pluripotent stem cell (iPSC)-derived cortical neurons, and SH-SY5Y neuroblastoma-derived neurons [33,34,86,126]. As these structures continue to be investigated and new preservation methods are developed, we speculate that cytonemes will be found in diverse TMEs and cancers.

Collectively, these studies establish several emerging principles of cytoneme biology in cancer. First, cytonemes facilitate transport of signaling pathways that are central drivers of tumor progression, including BMP, FGF, Notch, and WNT signaling, with this list likely to grow extensively in the near future with ongoing research. Second, cytoneme-mediated signaling occurs in both homotypic interactions across tumor cells and is heterotypical between tumors and the surrounding stroma. These events enable reciprocal interactions that shape the TME driving cancer progression. Importantly, targeting and disrupting cytoneme formation consistently suppresses tumor-associated signaling and reduces disease progression across all of these different cancer models [14,32,33,34].

## 8. Conclusions and Future Directions

The growing appreciation of cytoskeleton-derived cellular extensions has fundamentally altered how intercellular communication is viewed in cell and cancer biology. Rather than relying solely on diffusion-based gradients, tumors appear capable of establishing highly organized communication networks through specialized membrane protrusions that enable direct, targeted exchange of information between cancer cells and the TME. Evidence supporting functional roles for tunneling nanotubes, tumor microtubes, and cytonemes suggests that contact-mediated signaling is an underappreciated but potentially central mechanism driving tumor progression, therapeutic resistance, and microenvironmental remodeling [6,15,41,55].

Among these structures, cytonemes may represent a particularly important and previously overlooked signaling mechanism, specializing in the delivery and reception of signaling molecules. The fact that many developmental pathways implicated in cytoneme biology are also among the most prominent pathways reactivated in diverse cancers strongly suggests that cytoneme-mediated communication may be far more widespread in tumors than is currently appreciated. Consequently, cytoneme signaling may not represent a novel tumor-specific mechanism, but rather the reactivation or dysregulation of physiological signaling programs that normally regulate tissue organization and repair. This is supported by emerging studies in both *Drosophila* and human cancer models demonstrating that cytonemes can directly regulate tumor growth, invasion, stemness, and stromal reprogramming, raising the possibility that they constitute a conserved signaling infrastructure that tumors exploit to coordinate cancer progression.

A major challenge moving forward is determining the true prevalence of cytonemes in human cancers. Currently, there are many technical limitations to effectively visualize these delicate and dynamic extensions in postnatal tissues. Furthermore, overlap in nomenclature and morphology with filopodia, TNTs, and other extensions has introduced substantial ambiguity into the field [59]. Future research depends on the development of new methodologies that optimize for cytoneme detection in any intact tissues, including clinical specimens and tumor biopsies. Additionally, identifying specific molecular markers will dramatically aid in distinguishing cytonemes from TNTs and other extensions. This latter point may be more difficult to overcome, as it has become clear that different cellular extensions utilize many of the same core cytoskeletal proteins and signaling cascades to drive their elongation and maintenance [6]. Current approaches of characterizing extension morphology and molecular cargos may be sufficient, but a re-evaluation and clear consensus for the nomenclature of these cellular extensions may be required for a clearly defined set of parameters to be met for each structure.

Another important unresolved question is how cytonemes integrate into the broader landscape of tumor-associated cellular extensions. Stromal and tumor cells simultaneously generate multiple classes of membrane extensions, including filopodia, invadopodia, TNTs, and TMs, with overlapping cytoskeletal regulators [5,6,30,33,86]. There is currently insufficient evidence to conclude if tumors preferentially utilize cytonemes or other extension. Instead, these extensions likely coexist to serve distinct but complementary functions, with cytonemes specialized for ligand and receptor exchange, while TNTs and TMs facilitate the transfer of cytoplasmic cargo, organelles, and stress-response factors. This would allow simultaneous regulation of signaling, metabolism, survival, and invasion need for tumors and the TME to evolve and grow. Furthermore, whether cells actively transition between different protrusion states remains largely unknown. The substantial overlap in the molecular machinery controlling extension formation does suggest plasticity between states.

Regardless of classification ambiguity, the therapeutic potential for targeting TNTs and cytonemes to disrupt cancer signaling warrants further investigation. Targeting the cellular machinery responsible for their formation, maintenance, or cargo transport could simultaneously suppress several tumor-promoting processes, disrupting growth, migration, stemness, and invasion. Myo10 and other regulators of cytoneme assembly may therefore represent an emerging class of therapeutic targets. Evidence is clear that preventing contact-mediated communication between cancer cells and their microenvironment may be as effective as targeting individual signaling pathways.

A continuous growing body of evidence has shown that cancers exploit a diverse network of cytoskeletal extensions to establish communication channels beyond simple diffusion. While TNTs and tumor microtubes contribute to intercellular connectivity and stress adaptation, cytonemes are emerging as critical organizers of spatially restricted signaling within tumors. Defining the prevalence, molecular diversity, and functional significance of cytonemes in human cancers currently represents a major challenge for the field, but it is likely that cytoneme-mediated signaling will prove to be a widespread and therapeutically relevant component of cancer biology.

## Figures and Tables

**Table 1:** Cytoskeletal extensions in cancer.

	Major Cytoskeletal Composition	Morphology	Key Molecular Features	Primary Function in Cancer
Lamellipodia	Branched actin network [16].	Broad, sheet-like leading-edge protrusions [16,17].	Rac1-driven Arp2/3-dependent actin polymerization [18,19].	Cell migration, directional movement, and collective invasion [10,20].
Filopodia	Parallel actin bundles [21].	Thin, finger-like protrusions [21].	Cdc42 GTPase, Ena/VASP-driven, and formin-dependent [10,16].	Environmental sensing, cell guidance, adhesion, and metastatic dissemination [10,11,22,23]
Invadopodia	Actin-rich core, with microtubules [12].	Thick actin rich cellular protrusions, can form adhesions rings [12].	Concentrates and releases MMPs, including MT1-MMP, MMP2, and MMP9 [12,24,25].	Extracellular matrix degradation and tissue invasion [24,25].
Microtentacle	Predominantly microtubule-based [26].	Flexible extensions on circulating tumor cells [13].	Composed of linear and polarized α and β tubulin heterodimers. Vimentin stabilizes formation [26,27,28].	Reattachment of circulating tumor cells and metastatic seeding [13,27].
Cytoneme (Signaling Filopodia)	Parallel actin bundles [5].	Very thin, long closed-ended membrane extensions, carrying signaling molecules or receptors [5].	Cdc42 GTPase-driven. Myosin 10 is essential for formation, regulation, and cargo transport [29,30,31].	Direct cell–cell signaling and morphogen transport, promoting growth and stemness [14,32,33,34].
Tunneling nanotube (TNT)	Unbranched, and branched actin; can also contain microtubules [35,36].	Closed- or open-ended. Thin tubular bridges between cells [37].	Similar actin remodeling components as cytonemes and other extensions. Cytoplasmic transfer between cells with open-ended TNTs [5,6,38].	Transfer of organelles, nucleic acids, proteins, and drug-resistance factors for stress mitigation [39,40,41].
Tumor Microtube (TM)	Contains actin, but tubulin microtubules heavy, with myosin IIa [42].	Open-ended, thicker, and potentially longer than TNTs, frequently associated with gliomas [43].	Enriched with Gap43 and Cx43 [43,44].	Chemotherapy, radiotherapy, and surgical resistance, promoting tumor cell survival and invasion through similar mechanism as TNTs [42,44,45].

## Data Availability

No new data were created or analyzed in this study. Data sharing is not applicable to this article.

## References

[1] Wang Y., Zhou H., Ju S., Dong X., Zheng C. (2025). The solid tumor microenvironment and related targeting strategies: A concise review. Front. Immunol..

[2] Li Z., Li J., Bai X., Huang X., Wang Q. (2024). Tumor microenvironment as a complex milieu driving cancer progression: A mini review. Clin. Transl. Oncol..

[3] de Visser K.E., Joyce J.A. (2023). The evolving tumor microenvironment: From cancer initiation to metastatic outgrowth. Cancer Cell.

[4] Ritchie S., Reed D.A., Pereira B.A., Timpson P. (2021). The cancer cell secretome drives cooperative manipulation of the tumour microenvironment to accelerate tumourigenesis. Fac. Rev..

[5] Daly C.A., Hall E.T., Ogden S.K. (2022). Regulatory mechanisms of cytoneme-based morphogen transport. Cell. Mol. Life Sci..

[6] Liu S., Daly C.A., Ogden S.K., Zurzolo C. (2026). Regulation and function of specialized membrane protrusions in intercellular communication. Nat. Rev. Mol. Cell Biol..

[7] Ma J., Yu H., Yao S., Yan Y., Gu Z., Wang Z., Huang H., Chen D. (2025). Making cells inter-connected for signaling communication: A developmental view of cytonemes. Cell Commun. Signal..

[8] Zhang C., Scholpp S. (2019). Cytonemes in development. Curr. Opin. Genet. Dev..

[9] Kornberg T.B. (2014). Cytonemes and the dispersion of morphogens. Wiley Interdiscip. Rev. Dev. Biol..

[10] Pratiwi L., Elisa E., Sutanto H. (2024). Probing the protrusions: Lamellipodia and filopodia in cancer invasion and beyond. Mechanobiol. Med..

[11] Jacquemet G., Hamidi H., Ivaska J. (2015). Filopodia in cell adhesion, 3D migration and cancer cell invasion. Curr. Opin. Cell Biol..

[12] Linder S., Cervero P., Eddy R., Condeelis J. (2023). Mechanisms and roles of podosomes and invadopodia. Nat. Rev. Mol. Cell Biol..

[13] Kainka L., Shaebani R., Kaiser K., Bosche J., Santen L., Lautenschläger F. (2025). Microtubule polymerization generates microtentacles important in circulating tumor cell invasion. Biophys. J..

[14] Fereres S., Hatori R., Hatori M., Kornberg T.B. (2019). Cytoneme-mediated signaling essential for tumorigenesis. PLoS Genet..

[15] Pinto G., Brou C., Zurzolo C. (2020). Tunneling Nanotubes: The Fuel of Tumor Progression?. Trends Cancer.

[16] Innocenti M. (2018). New insights into the formation and the function of lamellipodia and ruffles in mesenchymal cell migration. Cell Adhes. Migr..

[17] SenGupta S., Parent C.A., Bear J.E. (2021). The principles of directed cell migration. Nat. Rev. Mol. Cell Biol..

[18] Ridley A.J., Paterson H.F., Johnston C.L., Diekmann D., Hall A. (1992). The small GTP-binding protein rac regulates growth factor-induced membrane ruffling. Cell.

[19] Weed S.A., Karginov A.V., Schafer D.A., Weaver A.M., Kinley A.W., Cooper J.A., Parsons J.T. (2000). Cortactin Localization to Sites of Actin Assembly in Lamellipodia Requires Interactions with F-Actin and the Arp2/3 Complex. J. Cell Biol..

[20] Carmona G., Perera U., Gillett C., Naba A., Law A.-L., Sharma V.P., Wang J., Wyckoff J., Balsamo M., Mosis F. (2016). Lamellipodin promotes invasive 3D cancer cell migration via regulated interactions with Ena/VASP and SCAR/WAVE. Oncogene.

[21] Blake T.C.A., Gallop J.L. (2023). Filopodia In Vitro and In Vivo. Annu. Rev. Cell Dev. Biol..

[22] Herman H., Fazakas C., Haskó J., Molnár K., Mészáros Á., Nyúl-Tóth Á., Szabó G., Erdélyi F., Ardelean A., Hermenean A. (2019). Paracellular and transcellular migration of metastatic cells through the cerebral endothelium. J. Cell. Mol. Med..

[23] Shibue T., Brooks M.W., Fatih Inan M., Reinhardt F., Weinberg R.A. (2012). The outgrowth of micrometastases is enabled by the formation of filopodium-like protrusions. Cancer Discov..

[24] Hao Z., Zhang M., Du Y., Liu J., Zeng G., Li H., Peng X. (2025). Invadopodia in cancer metastasis: Dynamics, regulation, and targeted therapies. J. Transl. Med..

[25] Leong H.S., Robertson A.E., Stoletov K., Leith S.J., Chin C.A., Chien A.E., Hague M.N., Ablack A., Carmine-Simmen K., McPherson V.A. (2014). Invadopodia are required for cancer cell extravasation and are a therapeutic target for metastasis. Cell Rep..

[26] Killilea A.N., Csencsits R., Le E.B.N.T., Patel A.M., Kenny S.J., Xu K., Downing K.H. (2017). Cytoskeletal Organization in Microtentacles. Exp. Cell Res..

[27] Matrone M.A., Whipple R.A., Balzer E.M., Martin S.S. (2010). Microtentacles tip the balance of cytoskeletal forces in circulating tumor cells. Cancer Res..

[28] Whipple R.A., Balzer E.M., Cho E.H., Matrone M.A., Yoon J.R., Martin S.S. (2008). Vimentin filaments support extension of tubulin-based microtentacles in detached breast tumor cells. Cancer Res..

[29] Stanganello E., Hagemann A.I.H., Mattes B., Sinner C., Meyen D., Weber S., Schug A., Raz E., Scholpp S. (2015). Filopodia-based Wnt transport during vertebrate tissue patterning. Nat. Commun..

[30] Hall E.T., Dillard M.E., Stewart D.P., Zhang Y., Wagner B., Levine R.M., Pruett-Miller S.M., Sykes A., Temirov J., E Cheney R. (2021). Cytoneme delivery of sonic hedgehog from ligand-producing cells requires myosin 10 and a dispatched-boc/cdon co-receptor complex. eLife.

[31] Koizumi K., Takano K., Kaneyasu A., Watanabe-Takano H., Tokuda E., Abe T., Watanabe N., Takenawa T., Endo T. (2012). RhoD activated by fibroblast growth factor induces cytoneme-like cellular protrusions through mDia3C. Mol. Biol. Cell.

[32] Boukhatmi H., Martins T., Pillidge Z., Kamenova T., Bray S. (2020). Notch Mediates Inter-tissue Communication to Promote Tumorigenesis. Curr. Biol..

[33] Rogers S., Zhang C., Anagnostidis V., Liddle C., Fishel M.L., Gielen F., Scholpp S. (2023). Cancer-associated fibroblasts influence Wnt/PCP signaling in gastric cancer cells by cytoneme-based dissemination of ROR2. Proc. Natl. Acad. Sci. USA.

[34] Routledge D., Rogers S., Ono Y., Brunt L., Meniel V., Tornillo G., Ashktorab H., Phesse T.J., Scholpp S. (2022). The scaffolding protein flot2 promotes cytoneme-based transport of wnt3 in gastric cancer. eLife.

[35] Goodman S., Naphade S., Khan M., Sharma J., Cherqui S. (2019). Macrophage polarization impacts tunneling nanotube formation and intercellular organelle trafficking. Sci. Rep..

[36] Henderson J.M., Ljubojevic N., Belian S., Chaze T., Castaneda D., Battistella A., Gianetto Q.G., Matondo M., Descroix S., Bassereau P. (2023). Tunnelling nanotube formation is driven by Eps8/IRSp53-dependent linear actin polymerization. EMBO J..

[37] Brou C., Zurzolo C. (2026). Building the bridges: Molecular mechanisms of tunneling nanotube formation. Cell. Mol. Life Sci..

[38] Rustom A., Saffrich R., Markovic I., Walther P., Gerdes H.-H. (2004). Nanotubular highways for intercellular organelle transport. Science.

[39] Haimovich G., Dasgupta S., Gerst J.E. (2021). RNA transfer through tunneling nanotubes. Biochem. Soc. Trans..

[40] Hekmatshoar Y., Nakhle J., Galloni M., Vignais M.-L. (2018). The role of metabolism and tunneling nanotube-mediated intercellular mitochondria exchange in cancer drug resistance. Biochem. J..

[41] Padmanabhan S., Deniz K., Sarkari A., Lou E. (2024). Tunneling Nanotubes: Implications for Chemoresistance. Results Probl. Cell Differ..

[42] Osswald M., Jung E., Sahm F., Solecki G., Venkataramani V., Blaes J., Weil S., Horstmann H., Wiestler B., Syed M. (2015). Brain tumour cells interconnect to a functional and resistant network. Nature.

[43] Wang X., Liang J., Sun H. (2022). The Network of Tumor Microtubes: An Improperly Reactivated Neural Cell Network with Stemness Feature for Resistance and Recurrence in Gliomas. Front. Oncol..

[44] Roehlecke C., Schmidt M.H.H. (2020). Tunneling Nanotubes and Tumor Microtubes in Cancer. Cancers.

[45] Weil S., Osswald M., Solecki G., Grosch J., Jung E., Lemke D., Ratliff M., Hänggi D., Wick W., Winkler F. (2017). Tumor microtubes convey resistance to surgical lesions and chemotherapy in gliomas. Neuro-Oncol..

[46] Wang X., Gerdes H.H. (2015). Transfer of mitochondria via tunneling nanotubes rescues apoptotic PC12 cells. Cell Death Differ..

[47] Ady J.W., Desir S., Thayanithy V., Vogel R.I., Moreira A.L., Downey R.J., Fong Y., Manova-Todorova K., Moore M.A.S., Lou E. (2014). Intercellular communication in malignant pleural mesothelioma: Properties of tunneling nanotubes. Front. Physiol..

[48] Lou E., Zhai E., Sarkari A., Desir S., Wong P., Iizuka Y., Yang J., Subramanian S., McCarthy J., Bazzaro M. (2018). Cellular and molecular networking within the ecosystem of cancer cell communication via tunneling nanotubes. Front. Cell Dev. Biol..

[49] Resnik N., Prezelj T., De Luca G.M.R., Manders E., Polishchuk R., Veranič P., Kreft M.E. (2018). Helical organization of microtubules occurs in a minority of tunneling membrane nanotubes in normal and cancer urothelial cells. Sci. Rep..

[50] Guan F., Wu X., Zhou J., Lin Y., He Y., Fan C., Zeng Z., Xiong W. (2024). Mitochondrial transfer in tunneling nanotubes—A new target for cancer therapy. J. Exp. Clin. Cancer Res..

[51] Ikeda H., Kawase K., Nishi T., Watanabe T., Takenaga K., Inozume T., Ishino T., Aki S., Lin J., Kawashima S. (2025). Immune evasion through mitochondrial transfer in the tumour microenvironment. Nature.

[52] Hoover G., Gilbert S., Curley O., Obellianne C., Lin M.T., Hixson W., Pierce T.W., Andrews J.F., Alexeyev M.F., Ding Y. (2025). Nerve-to-cancer transfer of mitochondria during cancer metastasis. Nature.

[53] Mokhles F., Moosavi M.A., Gutierrez-Uzquiza A., Velasco G., Li M., Cordani M. (2026). Unraveling stress-adaptation pathways in cancer: Functional dissection through CRISPR-based genetic screens. Cancer Lett..

[54] Valdebenito S., Malik S., Luu R., Loudig O., Mitchell M., Okafo G., Bhat K., Prideaux B., Eugenin E.A. (2021). Tunneling nanotubes, TNT, communicate glioblastoma with surrounding non-tumor astrocytes to adapt them to hypoxic and metabolic tumor conditions. Sci. Rep..

[55] Melwani P.K., Pandey B.N. (2023). Tunneling nanotubes: The intercellular conduits contributing to cancer pathogenesis and its therapy. Biochim. Biophys. Acta Rev. Cancer.

[56] Venkataramani V., Tanev D.I., Strahle C., Studier-Fischer A., Fankhauser L., Kessler T., Körber C., Kardorff M., Ratliff M., Xie R. (2019). Glutamatergic synaptic input to glioma cells drives brain tumour progression. Nature.

[57] Hombach-Klonisch S., Hall E., Amin R., Fedora E., Vriend J., Pitz M., Klonisch T. (2026). Neuroglial-Breast Cancer Crosstalk Shapes the Brain Metastatic Niche. Cells.

[58] Han X., Wang X. (2021). Opportunities and Challenges in Tunneling Nanotubes Research: How Far from Clinical Application?. Int. J. Mol. Sci..

[59] Dong X., Xuan Y., Wang M., Chang S., Hu X., Han Z. (2026). Tunneling nanotubes: A new dimension of intercellular communication and recent progress. Cell Commun. Signal..

[60] Roy S., Hsiung F., Kornberg T.B. (2011). Specificity of *Drosophila* cytonemes for distinct signaling pathways. Science.

[61] Kornberg T.B., Roy S. (2014). Cytonemes as specialized signaling filopodia. Development.

[62] Gustafson T., Wolpert L. (1961). Studies on the cellular basis of morphogenesis in the sea urchin embryo. Gastrulation in vegetalized larvae. Exp. Cell Res..

[63] Ramírez-Weber F.A., Kornberg T.B. (1999). Cytonemes: Cellular processes that project to the principal signaling center in *Drosophila* imaginal discs. Cell.

[64] Du L., Sohr A., Li Y., Roy S. (2022). GPI-anchored FGF directs cytoneme-mediated bidirectional contacts to regulate its tissue-specific dispersion. Nat. Commun..

[65] Patel A., Wu Y., Han X., Su Y., Maugel T., Shroff H., Roy S. (2022). Cytonemes coordinate asymmetric signaling and organization in the *Drosophila* muscle progenitor niche. Nat. Commun..

[66] Sato M., Kornberg T.B. (2002). FGF is an essential mitogen and chemoattractant for the air sacs of the *drosophila* tracheal system. Dev. Cell.

[67] Huang H., Kornberg T.B. (2015). Myoblast cytonemes mediate Wg signaling from the wing imaginal disc and Delta-Notch signaling to the air sac primordium. eLife.

[68] Zhang C., Brunt L., Ono Y., Rogers S., Scholpp S. (2023). Cytoneme-mediated transport of active Wnt5b–Ror2 complexes in zebrafish. Nature.

[69] Brunt L., Greicius G., Rogers S., Evans B.D., Virshup D.M., Wedgwood K.C.A., Scholpp S. (2021). Vangl2 promotes the formation of long cytonemes to enable distant Wnt/β-catenin signaling. Nat. Commun..

[70] Mattes B., Dang Y., Greicius G., Kaufmann L.T., Prunsche B., Rosenbauer J., Stegmaier J., Mikut R., Özbek S., Nienhaus G.U. (2018). Wnt/PCP controls spreading of Wnt/β-catenin signals by cytonemes in vertebrates. eLife.

[71] Hall E.T., Dillard M.E., Cleverdon E.R., Zhang Y., Daly C.A., Ansari S.S., Wakefield R., Stewart D.P., Pruett-Miller S.M., Lavado A. (2024). Cytoneme signaling provides essential contributions to mammalian tissue patterning. Cell.

[72] Greicius G., Mittermeier L., Liang R., Sigmundsson K., Chan Y.K., Liao P.-J., Ludwig A., Virshup D.M. (2025). Telocytes deliver essential Wnts directly to murine intestinal stem cells via synapse-like contacts. Dev. Cell.

[73] Clements R., Smith T., Cowart L., Zhumi J., Sherrod A., Cahill A., Hunter G.L. (2023). Myosin XV is a negative regulator of signaling filopodia during long-range lateral inhibition. Dev. Biol..

[74] Wang Y., Nguyen T., He Q., Has O., Forouzesh K., Eom D.S. (2025). Cytoneme-mediated intercellular signaling in keratinocytes is essential for epidermal remodeling in zebrafish. eLife.

[75] González-Méndez L., Gradilla A.C., Sánchez-Hernández D., González E., Aguirre-Tamaral A., Jiménez-Jiménez C., Guerra M., Aguilar G., Andrés G., Falcón-Pérez J.M. (2020). Polarized sorting of Patched enables cytoneme-mediated Hedgehog reception in the *Drosophila* wing disc. EMBO J..

[76] Gradilla A.C., González E., Seijo I., Andrés G., Bischoff M., González-Mendez L., Sánchez V., Callejo A., Ibáñez C., Guerra M. (2014). Exosomes as Hedgehog carriers in cytoneme-mediated transport and secretion. Nat. Commun..

[77] González-Méndez L., Seijo-Barandiarán I., Guerrero I. (2017). Cytoneme-mediated cell-cell contacts for Hedgehog reception. eLife.

[78] Sanders T.A., Llagostera E., Barna M. (2013). Specialized filopodia direct long-range transport of SHH during vertebrate tissue patterning. Nature.

[79] Zhang Z., Denans N., Liu Y., Zhulyn O., Rosenblatt H.D., Wernig M., Barna M. (2021). Optogenetic manipulation of cellular communication using engineered myosin motors. Nat. Cell Biol..

[80] Roy S., Huang H., Liu S., Kornberg T.B. (2014). Cytoneme-Mediated Contact-Dependent Transport of the *Drosophila* Decapentaplegic Signaling Protein. Science.

[81] Liang J., Pan Y., Yang J., Zeng D., Li J. (2025). WNT signaling in cancer: Molecular mechanisms and potential therapies. Mol. Biomed..

[82] Ehata S., Miyazono K. (2022). Bone Morphogenetic Protein Signaling in Cancer; Some Topics in the Recent 10 Years. Front. Cell Dev. Biol..

[83] Zhang H., Hang W., Jing Z.H., Liu B., Wang X., Li Y., Luo H., Lv H., Tao X., Timashev P. (2025). The role of notch signaling pathway in cancer: Mechanistic insights, therapeutic potential, and clinical progress. Front. Immunol..

[84] Cong G., Zhu X., Chen X.R., Chen H., Chong W. (2025). Mechanisms and therapeutic potential of the hedgehog signaling pathway in cancer. Cell Death Discov..

[85] Huang H., Liu S., Kornberg T.B. (2019). Glutamate signaling at cytoneme synapses. Science.

[86] Hall E., Ogden S. (2018). Preserve Cultured Cell Cytonemes through a Modified Electron Microscopy Fixation. Bio-Protocol.

[87] Hall E.T., Daly C.A., Zhang Y., Dillard M.E., Ogden S.K. (2022). Fixation of Embryonic Mouse Tissue for Cytoneme Analysis. JoVE J. Vis. Exp..

[88] Rogers S., Scholpp S. (2021). Preserving Cytonemes for Immunocytochemistry of Cultured Adherent Cells. Methods Mol. Biol..

[89] Bodeen W.J., Marada S., Truong A., Ogden S.K. (2017). A fixation method to preserve cultured cell cytonemes facilitates mechanistic interrogation of morphogen transport. Development.

[90] Lee K., Gallop J.L., Rambani K., Kirschner M.W. (2010). Self-assembly of filopodia-like structures on supported lipid bilayers. Science.

[91] Callejo A., Bilioni A., Mollica E., Gorfinkiel N., Andrés G., Ibáñez C., Torroja C., Doglio L., Sierra J., Guerrero I. (2011). Dispatched mediates Hedgehog basolateral release to form the long-range morphogenetic gradient in the *Drosophila* wing disk epithelium. Proc. Natl. Acad. Sci. USA.

[92] Sutton G., Brunt L., Bamsey J., Hernández-Huertas L., Sears E., Beaumont E., Patterson G., Liddle C., Chen Y., Moreno-Mateos M.A. (2026). Rab8a-positive vesicles transport Wnt8a along cytonemes in zebrafish embryogenesis. bioRxiv.

[93] Berg J.S., Cheney R.E. (2002). Myosin-X is an unconventional myosin that undergoes intrafilopodial motility. Nat. Cell Biol..

[94] Kerber M.L., Cheney R.E. (2011). Myosin-X: A MyTH-FERM myosin at the tips of filopodia. J. Cell Sci..

[95] Courson D.S., Cheney R.E. (2015). Myosin-X and disease. Exp. Cell Res..

[96] Rechsteiner M., Rogers S.W. (1996). PEST sequences and regulation by proteolysis. Trends Biochem. Sci..

[97] Gousset K., Marzo L., Commere P.H., Zurzolo C. (2013). Myo10 is a key regulator of TNT formation in neuronal cells. J. Cell Sci..

[98] Umeki N., Jung H.S., Sakai T., Sato O., Ikebe R., Ikebe M. (2011). Phospholipid-dependent regulation of the motor activity of myosin, X. Nat. Struct. Mol. Biol..

[99] Plantard L., Arjonen A., Lock J.G., Nurani G., Ivaska J., Strömblad S. (2010). PtdIns(3,4,5)P_3_ is a regulator of myosin-X localization and filopodia formation. J. Cell Sci..

[100] Weber K.L., Sokac A.M., Berg J.S., Cheney R.E., Bement W.M. (2004). A microtubule-binding myosin required for nuclear anchoring and spindle assembly. Nature.

[101] Fitz G.N., Weck M.L., Bodnya C., Perkins O.L., Tyska M.J. (2023). Protrusion growth driven by myosin-generated force. Dev. Cell.

[102] Xu J., Li Y., Novak C., Lee M., Yan Z., Bang S., McGinnis A., Chandra S., Zhang V., He W. (2026). Mitochondrial transfer from glia to neurons protects against peripheral neuropathy. Nature.

[103] Liu R., Woolner S., Johndrow J.E., Metzger D., Flores A., Parkhurst S.M. (2008). Sisyphus, the *Drosophila* myosin XV homolog, traffics within filopodia transporting key sensory and adhesion cargos. Development.

[104] Pozo F.M., Geng X., Tamagno I., Jackson M.W., Heimsath E.G., Hammer J.A., Cheney R.E., Zhang Y. (2021). MYO10 drives genomic instability and inflammation in cancer. Sci. Adv..

[105] Cao R., Chen J., Zhang X., Zhai Y., Qing X., Xing W., Zhang L., Malik Y.S., Yu H., Zhu X. (2014). Elevated expression of myosin X in tumours contributes to breast cancer aggressiveness and metastasis. Br. J. Cancer.

[106] Tokuo H., Bhawan J., Coluccio L.M. (2018). Myosin X is required for efficient melanoblast migration and melanoma initiation and metastasis. Sci. Rep..

[107] Arjonen A., Kaukonen R., Mattila E., Rouhi P., Högnäs G., Sihto H., Miller B.W., Morton J.P., Bucher E., Taimen P. (2014). Mutant p53-associated myosin-X upregulation promotes breast cancer invasion and metastasis. J. Clin. Investig..

[108] Peuhu E., Jacquemet G., Scheele C.L.G.J., Isomursu A., Laisne M.-C., Koskinen L.M., Paatero I., Thol K., Georgiadou M., Guzmán C. (2022). MYO10-filopodia support basement membranes at pre-invasive tumor boundaries. Dev. Cell.

[109] He J.-H., Chen J.-G., Zhang B., Chen J., You K.-L., Hu J.-M., Xu J.-W., Chen L. (2020). Elevated MYO10 Predicts Poor Prognosis and its Deletion Hampers Proliferation and Migration Potentials of Cells Through Rewiring PI3K/Akt Signaling in Cervical Cancer. Technol. Cancer Res. Treat..

[110] Ross M.E., Zhou X., Song G., Shurtleff S.A., Girtman K., Williams W.K., Liu H.-C., Mahfouz R., Raimondi S.C., Lenny N. (2003). Classification of pediatric acute lymphoblastic leukemia by gene expression profiling. Blood.

[111] Sun Y., Ai X., Shen S., Lu S. (2015). NF-κB-mediated miR-124 suppresses metastasis of non-small-cell lung cancer by targeting MYO10. Oncotarget.

[112] Dvornikov D., Schneider M.A., Ohse S., Szczygieł M., Titkova I., Rosenblatt M., Muley T., Warth A., Herth F.J., Dienemann H. (2018). Expression ratio of the TGFβ-inducible gene MYO10 is prognostic for overall survival of squamous cell lung cancer patients and predicts chemotherapy response. Sci. Rep..

[113] Makowska K.A., Hughes R.E., White K.J., Wells C.M., Peckham M. (2015). Specific Myosins Control Actin Organization, Cell Morphology, and Migration in Prostate Cancer Cells. Cell Rep..

[114] Kenchappa R.S., Mistriotis P., Wisniewski E., Bhattacharya S., Kulkarni T., West R., Luu A., Conlon M., Heimsath E., Crish J.F. (2020). Myosin 10 Regulates Invasion, Mitosis, and Metabolic Signaling in Glioblastoma. iScience.

[115] Schoumacher M., Goldman R.D., Louvard D., Vignjevic D.M. (2010). Actin, microtubules, and vimentin intermediate filaments cooperate for elongation of invadopodia. J. Cell Biol..

[116] Ou H., Wang L., Xi Z., Shen H., Jiang Y., Zhou F., Liu Y., Zhou Y. (2022). MYO10 contributes to the malignant phenotypes of colorectal cancer via RACK1 by activating integrin/Src/FAK signaling. Cancer Sci..

[117] Fusco N., Sajjadi E., Venetis K., Gaudioso G., Lopez G., Corti C., Rocco E.G., Criscitiello C., Malapelle U., Invernizzi M. (2020). PTEN Alterations and Their Role in Cancer Management: Are We Making Headway on Precision Medicine?. Genes.

[118] Yu H., Wang N., Ju X., Yang Y., Sun D., Lai M., Cui L., Sheikh M.A., Zhang J., Wang X. (2012). PtdIns (3,4,5) P3 Recruitment of Myo10 Is Essential for Axon Development. PLoS ONE.

[119] Yim Y.I., Pedrosa A., Wu X., Chinthalapudi K., Cheney R.E., Hammer J.A. (2023). Mechanisms underlying Myosin 10′s contribution to the maintenance of mitotic spindle bipolarity. Mol. Biol. Cell.

[120] Sandquist J.C., Larson M.E., Hine K.J. (2016). Myosin-10 independently influences mitotic spindle structure and mitotic progression. Cytoskeleton.

[121] Woolner S., O’Brien L.L., Wiese C., Bement W.M. (2008). Myosin-10 and actin filaments are essential for mitotic spindle function. J. Cell Biol..

[122] Toyoshima F., Nishida E. (2007). Integrin-mediated adhesion orients the spindle parallel to the substratum in an EB1- and myosin X-dependent manner. EMBO J..

[123] Bennett R.D., Mauer A.S., Pittelkow M.R., Strehler E.E. (2009). Calmodulin-like protein upregulates myosin-10 in human keratinocytes and is regulated during epidermal wound healing in vivo. J. Investig. Dermatol..

[124] Heimsath E.G., Yim Y.I., Mustapha M., Hammer J.A., Cheney R.E. (2017). Myosin-X knockout is semi-lethal and demonstrates that myosin-X functions in neural tube closure, pigmentation, hyaloid vasculature regression, and filopodia formation. Sci. Rep..

[125] Kast D.J., Yang C., Disanza A., Boczkowska M., Madasu Y., Scita G., Svitkina T., Dominguez R. (2014). Mechanism of IRSp53 inhibition and combinatorial activation by Cdc42 and downstream effectors. Nat. Struct. Mol. Biol..

[126] Piers T.M., Fang K., Namboori S.C., Liddle C., Rogers S., Bhinge A., Killick R., Scholpp S. (2024). WNT7A-positive dendritic cytonemes control synaptogenesis in cortical neurons. Development.

